# Direct characterization of solute transport in unsaturated porous media using fast X-ray synchrotron microtomography

**DOI:** 10.1073/pnas.2011716117

**Published:** 2020-09-08

**Authors:** Sharul Hasan, Vahid Niasar, Nikolaos K. Karadimitriou, Jose R. A. Godinho, Nghia T. Vo, Senyou An, Arash Rabbani, Holger Steeb

**Affiliations:** ^a^Department of Chemical Engineering and Analytical Science, University of Manchester, Manchester M13 9PL, United Kingdom;; ^b^Institute of Applied Mechanics, University of Stuttgart, 70569 Stuttgart, Germany;; ^c^Helmholtz-Zentrum Dresden-Rossendorf, Helmholtz Institute Freiberg for Resource Technology, 09599 Freiberg, Germany;; ^d^Diamond House, Didcot OX11 0DE, United Kingdom

**Keywords:** porous media, X-ray imaging, dispersion, two-phase flow, pore scale

## Abstract

Solute transport in unsaturated porous materials is a complex process, which exhibits some distinct features differentiating it from transport under saturated conditions. These features emerge mostly due to the different transport time scales at different regions of the flow network, which can be classified into flowing and stagnant regions, predominantly controlled by advection and diffusion, respectively. Under unsaturated conditions, the solute breakthrough curves show early arrivals and very long tails, and this type of transport is usually referred to as non-Fickian. This study directly characterizes transport through an unsaturated porous medium in three spatial dimensions at the resolution of 3.25 μm and the time resolution of 6 s. Using advanced high-speed, high-spatial resolution, synchrotron-based X-ray computed microtomography (sCT) we obtained detailed information on solute transport through a glass bead packing at different saturations. A large experimental dataset (>50 TB) was produced, while imaging the evolution of the solute concentration with time at any given point within the field of view. We show that the fluids’ topology has a critical signature on the non-Fickian transport, which yet needs to be included in the Darcy-scale solute transport models. The three-dimensional (3D) results show that the fully mixing assumption at the pore scale is not valid, and even after injection of several pore volumes the concentration field at the pore scale is not uniform. Additionally, results demonstrate that dispersivity is changing with saturation, being twofold larger at the saturation of 0.52 compared to that at the fully saturated domain.

## Significance

Solute transport in porous materials is pertinent to many engineering and industrial applications. This research provides direct characterization of spatiotemporal behavior of solute transport in saturated and unsaturated porous media using high-resolution four-dimensional synchrotron X-ray imaging. This research has two key impacts: 1) we demonstrate a workflow for direct characterization of solute transport in any porous material, which allows better design of fabricated porous materials and detailed characterization of natural porous materials, and 2) we evaluate the underlying assumptions used in the existing theories and identify the major gaps in theories that need to be addressed for better predictive modeling.

Solute transport in porous media is an important physical process that can be found in many industrial and natural systems. Fertilizer leaching in soils (1), contaminant transport in the groundwater and vadose zone (2), injection of chemicals to enhance oil recovery such as low-salinity water flooding (3), remediation of nonaqueous phase liquid-contaminated soils using surfactant flooding (4), and transport of ions in redox flow batteries (5, 6) are a few of the abundant examples of solute transport in partially saturated porous materials. Understanding transport in partially saturated porous materials is challenging due to the complex dynamics of two-phase flow and intricate fluid networks that control the interplay between the temporal and spatial transport characteristics (7–9). Transport on the pore scale is characterized by advection and diffusion. The ratio between advective and diffusive transport defines the dimensionless Péclet number, as a means to delineate the relative importance of these two transport mechanisms. However, on the Darcy scale, where pore space properties (e.g., porosity, saturation, and permeability) are averaged, advective transport leads to solute dispersion as a consequence of tortuous and spatially variable flow pathways in porous media. This becomes even more complex under two-phase flow conditions, as well as in heterogeneous (fractured) porous media, since the available pore space does not homogeneously contribute to flow; some regions are dead ends or very slow in transport due to poor connectivity to the main flow pathways. Thus, for a partially saturated porous medium, and depending on the flow conditions and the pore space connectivity, some regions are diffusion-dominated, while other regions can be advection-controlled. For increasing Péclet numbers, when the contrast between the two process-dependent time scales becomes significant, transport of solutes becomes increasingly complex and results in anomalous (non-Gaussian or non-Fickian) behavior, which cannot be captured by the classical advection–dispersion equation, as reported in many experiments (10–14).

In the case of single-phase transport in heterogeneous porous media, the space available to the fluids does not change with time (15, 16). Thus, the non-Fickian behavior is a function of the pore morphology (17) and the Péclet number (18). However, under two-phase flow conditions the saturation topology can change due to the interplay between viscous and capillary forces (19). Thus, the non-Fickian behavior also depends on the fluids’ topology (20, 21). Due to the direct impact of two-phase flow and fluid topology on the spatiotemporal characteristics of transport, it is important to integrate the two-phase flow theories with the theories developed for solute transport in partially saturated porous media. However, currently, there is no fully integrated Darcy-scale theory that captures both two-phase flow and transport for partially saturated porous media. None of the commonly used theories, including the mobile–immobile model (MIM) (22), the multirate mass transfer (MRMT) (23), and continuous time random walk (24) models include two-phase flow formulations in their theoretical developments. To date, the capability of such models to match the experimental data using inverse modeling has been tested, but the physical consistency of such models is still open to be evaluated (25, 26).

In an effort to improve our understanding of the fundamental physical processes occurring during two-phase flow and transport, this paper has three main objectives which have been achieved and are reported: 1) an imaging technique has been established to evaluate the time-resolved three-dimensional (3D) concentration fields in a partially saturated porous medium, 2) a unique dataset has been generated that can be used for validation of the existing theories of transport in unsaturated porous media, and 3) insights into the effect of the pore-scale fluids topology on the non-Fickian transport in a 3D system are provided.

These three achievements have been materialized using high spatial and temporal resolution, synchrotron-based, X-ray computed microtomography (sCT) at beamline I12 (27), Diamond Light Source, United Kingdom, to produce a time-resolved, 3D concentration field with a time resolution of 6 s and the spatial resolution of 3.25 μm of a partially saturated glass bead packing. The process of capturing the concentration of a solute in 3D space as a function of time is commonly referred to as 4D sCT, where the fourth dimension is time. The 4D sCT experiments produced a massive dataset (>50 TB) which required intensive and tedious postprocessing and an evaluation process as explained in *SI Appendix*. As a result of the postprocessing, the local change of concentration at different steady-state saturations and Péclet numbers was visualized. Consequently, we were able to provide the evidence that fluids’ topology is the key characteristic, which needs to be addressed in the effort to increase the predictive capability of the existing theories of non-Fickian transport in unsaturated porous media. Also, in a 3D porous medium, we managed to capture the non-Fickian transport by monitoring the solute’s resident concentration and show that the non-Fickian behavior is a fundamental characteristic and is present even on the pore scale. This is due to the separation of streamlines at the pore scale and significant variation of the velocity across the cross section. Former studies at 2D regular micromodels (28) showed that there was not a full mixing condition at pore bodies. To conclude, we also quantified the dispersivity of the packing (which represents the length scale at which significant dispersion takes place) (29) based on the resident concentration on the pore scale and also linked it to the global saturation and the Péclet number.

## Determination of the Average Resident Concentration

The glass beads porous medium was initially fully saturated with the nonwetting phase (Fluorinert FC-43). Then, primary imbibition was performed by injecting pure water at a given flow rate into the system to establish a steady-state saturation topology. After injection of many pore volumes to reach the steady-state saturation, a potassium iodine (KI) aqueous solution was injected at a constant rate into the medium to disperse it inside the space initially filled with water. Due to the presence of iodine ions, which served as the solute, there was an attenuation factor for the X-rays coming from the source (30) creating a contrast in the corresponding images according to its concentration. The sCT experiments captured the concentration field of the KI solution every 6 s, and this capturing process can be broken down to two major processes. The acquisition of a full stack of tomographs, meaning a complete scan of the sample, took 3 s, with the rotation of the sample back to the original position taking another 3 s. The captured images were reconstructed using an in-house data processing pipeline that was written in Python. The process to reconstruct the captured image included a flat-field correction (for the removal of the background noise), zinger removal which is a process for removing the image artifact in the form of bright straight or zinger, ring artifact removal (31), denoising, automated determination of the center of rotation (32), and filtered-back projection reconstruction (33, 34). At a later stage, the reconstructed image were rescaled from 32-bit to 8-bit in order to reduce the computation time of image filtering and image segmentation.

As the KI solution was injected into the medium, it gradually mixed with water. As a result, the grayscale intensity in each voxel, initially having a low grayscale intensity value, shifted toward a higher grayscale intensity. The change in the grayscale value of a given voxel is related to the change of the local resident concentration of the iodine ions. In order to quantify this change, we needed to establish a correlation between the grayscale values and the actual KI concentration. Thus, individual experiments were performed in a fully saturated porous medium at five different known KI solution concentrations under steady-state conditions to establish the correlation function (*SI Appendix*, Fig. S4). Known concentrations of KI solutions were introduced into the water-saturated porous medium, and after the KI concentration had reached steady-state homogeneous conditions, meaning that KI solution is homogeneous distributed in the pore space, the average grayscale value was calculated. This was repeated for five known KI concentration values, and a linear trend between the concentration and grayscale values was found as shown in *SI Appendix*, Fig. S4, $c_{i}=aI_{i}+b$, where $c_{i}$ is the local resident concentration in voxel i in mol/L, with a and b serving as fitting coefficients. $I_{i}$ is the grayscale value in voxel i, and i is the index of a voxel in the 3D space, which is filled with pure water or KI solution. After converting the grayscale intensity to the local resident concentration, it was averaged over the total number of voxels belonging to the water and KI solution domain, to obtain the average concentration in the imaged section $C_{r}=\sum_{1}^{N}c_{i}/N$, where $C_{r}$ is the average resident concentration and N is the total number of voxels belonging to the pore space. The equations $c_{i}=aI_{i}+b$ and $C_{r}=\sum_{1}^{N}c_{i}/N$ were evaluated at different time steps where at a given time a specific state of solute transport was imaged by the sCT. Therefore, $C_{r}$ was plotted against time to generate the average resident concentration curves.

## Results and Discussions

### Non-Fickian Characteristics of Transport on the Pore Scale.

**Fig. 1.**
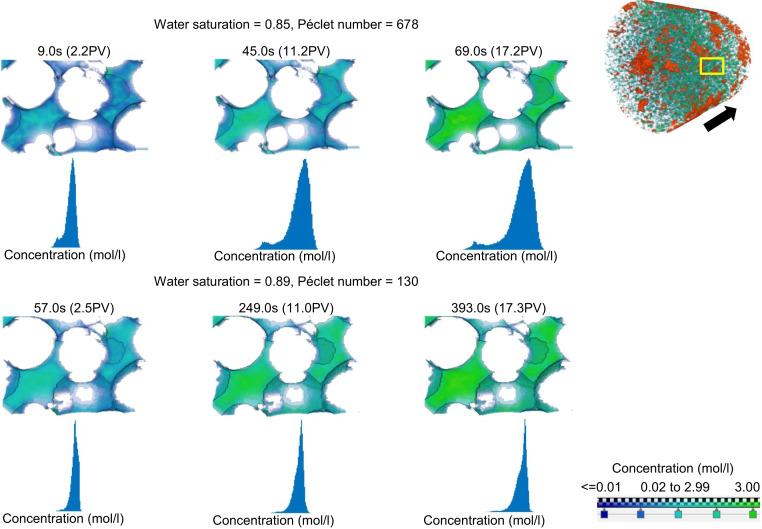
Concentration field and histogram of the concentration field for (*Top*) a water saturation of 0.85 with a Péclet number of 638 and (*Bottom*) a water saturation of 0.89 with a Péclet number of 130 at three different times and pore volumes. The pore volume is shown in parentheses besides time. The visible color spectrum shows the concentration field representing the concentration value. The concentration value for the visible color spectrum is illustrated in the legend located at the bottom right. The concentration field and the histogram of the concentration field for both cases of water saturation are located within the yellow box as illustrated in the top right corner with the black arrow indicating the mean flow path. The coefficient of variation of the first case is 2.5 times larger than the second case for the same injected pore volume.

As mentioned earlier, the characterization of transport in porous media is done based on the Péclet number. The Péclet number can be defined on both the macroscale (Darcy scale) and the microscale (pore scale). Based on the macroscale definition of Péclet number, $Pe=\frac{Q}{\phi SA}\times \frac{L}{D}$, where Q is the volumetric injection rate (μm^3^/s), S is the water saturation, A is the cross-sectional area of the medium (μm^2^), L is the length of the visualized domain (μm), and D is the molecular diffusion coefficient (μm^2^/s), which is 1.80 $\times 10^{-9}\;m^{2}$/s (35) for the diffusion of KI in water. Porosity (ϕ) is 0.24, and permeability is 4.5 darcy. To illustrate the impact of Péclet number on transport, we have imaged transport under two similar water saturations (0.85 and 0.89) but different Péclet numbers. Fig. 1 and *SI Appendix*, Fig. S3, show the two-phase configuration of the imaged section of the water-wet sample. Red color represents the nonwetting phase with green and blue colors representing the regions filled with the KI solution and water, respectively. To illustrate transport at the pore level, 3D concentration fields of the selected region (yellow box shown on the right in Fig. 1) are illustrated for two different macroscopic Péclet numbers at different times. The dimensionless time, indicated by the injected volume divided by the pore volume filled with water (PV), is shown for each figure. As it can be found from the images and the histogram of the pore-scale concentrations at two different Péclet numbers but the same saturation, transport is non-Fickian even at the pore scale. There might be two major reasons that cause a nonuniform concentration distribution even after several injected pore volumes: 1) significant segregation of streamlines within individual pore bodies and 2) significant discrepancy in the velocity field across the pores, as the transport close to the solid wall is very slow, while it is faster in the middle of the pores. To further investigate the cause of very slow mixing in pores, we conducted high-resolution lattice Boltzmann simulations to solve the velocity field. For the case of saturation of 0.89, we performed GPU-accelerated volumetric lattice Boltzmann method (36, 37). *SI Appendix*, Fig. S5 *A* and *B*, show the streamlines at two different scales for the same sample. The pore-scale streamlines (*SI Appendix*, Fig. S5*B*) clearly show that there is not a complete mixing at pore bodies, and the concentrations within the pore bodies are supplied from different upstream pores. The separation of the streamlines within the pore throats is also visible given that the pore throats are short, and there is not enough mixing length. Similar observation was reported in the 2D micromodel study (28). Additionally, the very slow velocities near wall and the fast transport along other streamlines lead to a nonuniform distribution of concentration across the cross section due to significant transport time scales across the pores. This is particularly visible for larger Péclet numbers as the discrepancy in the transport time scales for the area in the middle versus that close to the grain walls is significantly larger. The images for the larger Péclet number show that even after several pore volumes of KI solution injection, the concentration in the tight regions or close to the grain walls is low. As a result of the non-Fickian transport within the pore for the high Péclet number, a bimodal distribution of concentration was obtained, as shown in the concentration histograms in Fig. 1. Note that even after 17 PV injection, the concentration in the domain chosen is still bimodal for a Péclet number of 678. The small and large peaks represent the low and high concentrations, respectively. In contrast to the numerous simulation studies which assume fully mixing conditions in the pores (38–41), these insights into the pore-level transport in porous media provide the evidence that the assumption of the fully mixed concentration is not valid, and the error induced by this assumption would increase with increasing the Péclet number.

**Fig. 2.**
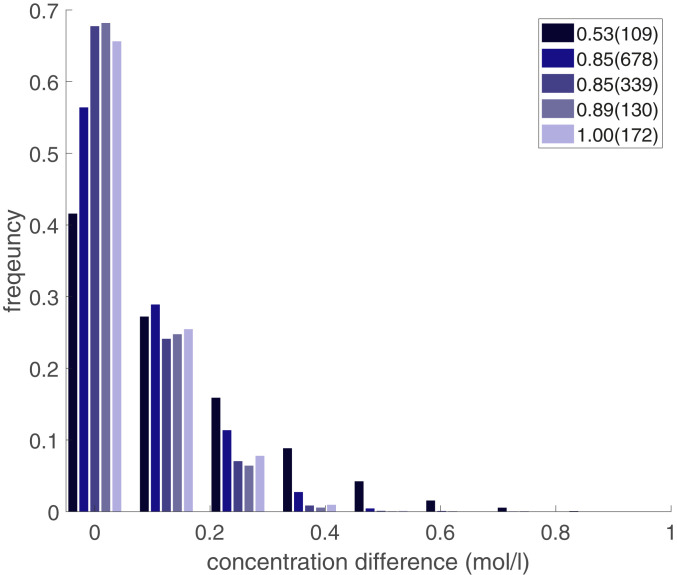
Histogram of the concentration difference for water saturation of 0.53, 0.85, 0.89, and 1.00. Each saturation case has a different Péclet number as illustrated in parentheses in the legend. The concentration difference is defined as $\Delta c=\max\left\{\left|c_{i}-c_{j}\right|\right\}$, the concentration difference between two neighboring voxels. All of the plotted cases were analyzed at the average resident concentration of 2.5 mol/L.

Another important factor is to illustrate the non-Fickian transport on the macroscale at different saturations and Péclet numbers. In several 2D micromodel experiments it has been shown that under two-phase flow conditions, some regions are stagnant or very slow (immobile), and some regions are flowing (mobile) (7, 9, 42). Given the fact that wettability controls the film and corner flow, and this porous medium is water-wet, the injected water can cover the surface of grains. That indicates that the probability of the stagnant saturation is smaller compared to the case of a hydrophobic porous medium. Our preliminary analysis of the 4D sCT for a given water saturation indicated that there is no evidence of presence of completely stagnant regions in this porous medium due to the water-wet status of beads. To demonstrate the change in the transport time scale at different saturations and Péclet numbers, we estimated the KI concentration difference in the pore space filled with water or the KI aqueous solution for neighboring voxels, based on the expectation that at the interface between the fast and slow regions the concentration difference should be larger than the case of fully flowing (mobile) condition. Fig. 2 shows the distribution of the spatial concentration difference for each two neighboring voxels at saturations of 0.53, 0.85, 0.89, and 1.00 and five different Péclet numbers. The average resident concentrations in all these five cases are the same and equal to 2.5 mol/L (83% of maximum). Péclet numbers are shown for each saturation as well, within parentheses in the legend. While the saturation of 0.53 has a small Péclet number and is expected to be less heterogeneously transported, it shows a clearly skewed distribution of local concentration difference. That indicates the major impact of fluids topology. For the saturation cases of 0.85 to 0.89 at three distinct Péclet numbers, we can conclude that with increase of flow rate the discrepancy between the slow and fast regions increases, which agrees with the literature (7, 22). Based on these local spatial concentration fields, we can conclude that saturation topology plays a much stronger role in non-Fickian transport compared to the Péclet number.

### Signature of Water Saturation on Solute Transport.

**Fig. 3.**
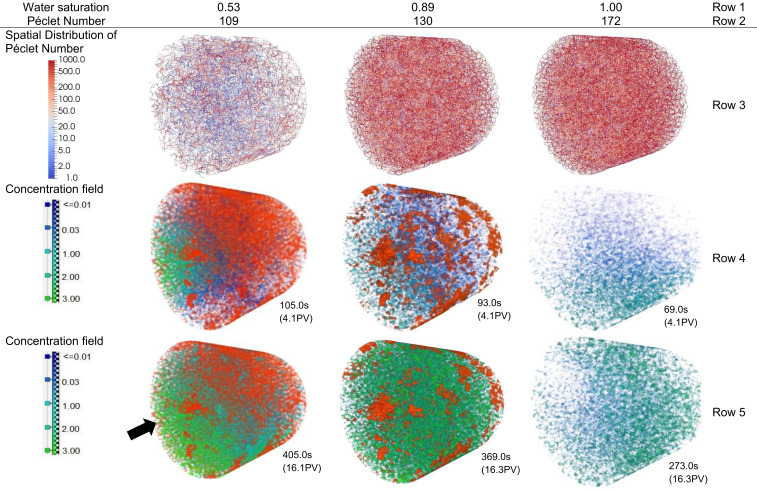
Row 1 represents water saturation. Row 2 represents the Péclet number for each water saturation case. Row 3 represents the spatial distribution of the computed local Péclet number in the extracted pore network. Rows 4 and 5 are the concentration fields at the time shown below the images. The mean flow path for the concentration field is from the left side to the right side of the image (black arrow). The red color fluid shows the nonwetting phase (Fluorinert), and green to blue color spectrum shows the variation in the KI solution concentration from the high concentration (3 mol/L) to the low concentration (0.01 mol/L), respectively (56).

In the previous section of this article we showed that at similar water saturations, the Péclet number significantly influenced the solute spreading in the flowing zone on the pore scale, which was also reported from the breakthrough curves in former experiments (10–14). In this section, we demonstrate the effect of water saturation on average resident concentration. However, in order to establish different degrees of saturation, the injection rate and corresponding Péclet number needed to be increased. The experimental results for water saturations of 0.53, 0.89, and 1 (and macroscopic Péclet numbers of 109, 130, and 172, respectively) are presented in Fig. 3. Also, two concentration fields per each saturation topology are shown. The small variability in the macroscopic Péclet numbers indicates the small variation in the advective transport at these three different saturations.

**Fig. 4.**
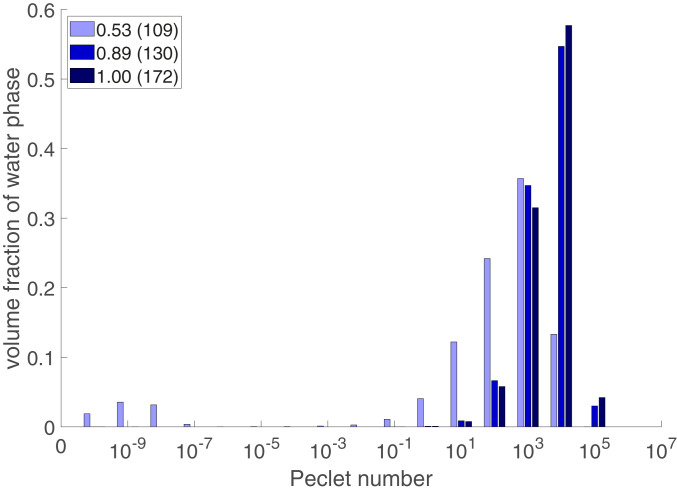
Histogram of the pore-scale Péclet number for the water saturations of 0.53, 0.89, and 1.00. Water saturation of 0.53 has a macroscopic Péclet number of 109, while water saturations of 0.89 and 1.00 have Péclet numbers of 130 and 172, respectively. The *y*-axis values are calculated as the total of volume of pore throats that filled with water belonging to a Péclet number bin divided by the total of volume of pore throats filled with water ($\sum_{Pe_{i}}r_{ij}^{2}l_{ij}/\sum r_{ij}^{2}l_{ij}$).

To evaluate the variation of the pore-scale Péclet number, the experimental data were amended with the pore network simulations, as the direct evaluation of the pore-scale velocity was out of the capability of the X-ray imaging method. Results from the sCT experiments were postprocessed to segment the water and Fluorinert network topology within the pore space to extract the pore network. The pressure field in the water-filled pore network was computed assuming Hagen–Poiseuille flow (41), with the assigned boundary conditions being similar to those of the experiments. Using the local pore velocity, the local Péclet number in each pore throat was calculated, given by $Pe_{ij}=v_{ij}r_{ij}/D_{m}$ (microscale definition of the Peclet number) where $v_{ij}$ is the average velocity in the pore throat ij, $r_{ij}$ is the radius of the pore throat ij, and $D_{m}$ is the molecular diffusion coefficient, set to 1.80 $\times 10^{-9}\;m^{2}$/s (35). To better visualize this variability of the local Péclet number, the histogram of the local Péclet number was created for water saturations of 0.53, 0.89, and 1.00 (Fig. 4). The *y* axis of the histogram is defined as the volume of pore throats, filled with water belonging to a Péclet number bin divided by the total volume of pore throats filled with water. Clearly, the range of the pore-scale Péclet at saturation of 0.53 is much broader than the fully saturated case (Fig. 3, row 3).

According to Fig. 4, for water saturation of 0.53, the histogram shows a clear bimodal distribution with the first small peak at $10^{-9}$ and a second distribution peak at $10^{3}$. This means that the transport of KI solution is influenced by two regions with two different transport time scales. The very small local Péclet numbers (first distribution peak at $10^{-9}$) correspond to the stagnant/slow regions in the water-saturated area, mostly diffusion controlled. On the other hand, the high local Péclet numbers (second distribution peak at $10^{3}$) correspond to the flowing regions, where transport is controlled by advection. Given that the small peak (stagnant regions) are statistically not significant, it justifies the reason that the completely stagnant regions were not found in the water-wet glass bead packing of this study. As the water saturation approaches unity, the distribution of the local Péclet number follows the normal distribution with the distribution of the local Péclet number reaching a peak value of $10^{4}$ which corresponds to advection-driven transport, indicating that the majority of the pore space is advective controlled with similar transport time scales.

Due to the presence of slow and fast regions, depending on the saturation topology as well as the microscale Péclet number, the concentration fields can significantly vary as shown in Fig. 3 (rows 4 and 5). At a water saturation of 0.53, the solute is transported through a preferential flow path to reach the outlet at time equal to 105.0 s. After that, the solute is diffused into the slow regions (Fig. 3, row 5, water saturation of 0.53). This dual transport behavior is known as non-Gaussian (non-Fickian) transport and has been reported based on effluent concentration measurements in partially saturated as well as single-phase heterogeneous or fractured porous media (43–46).

**Fig. 5.**
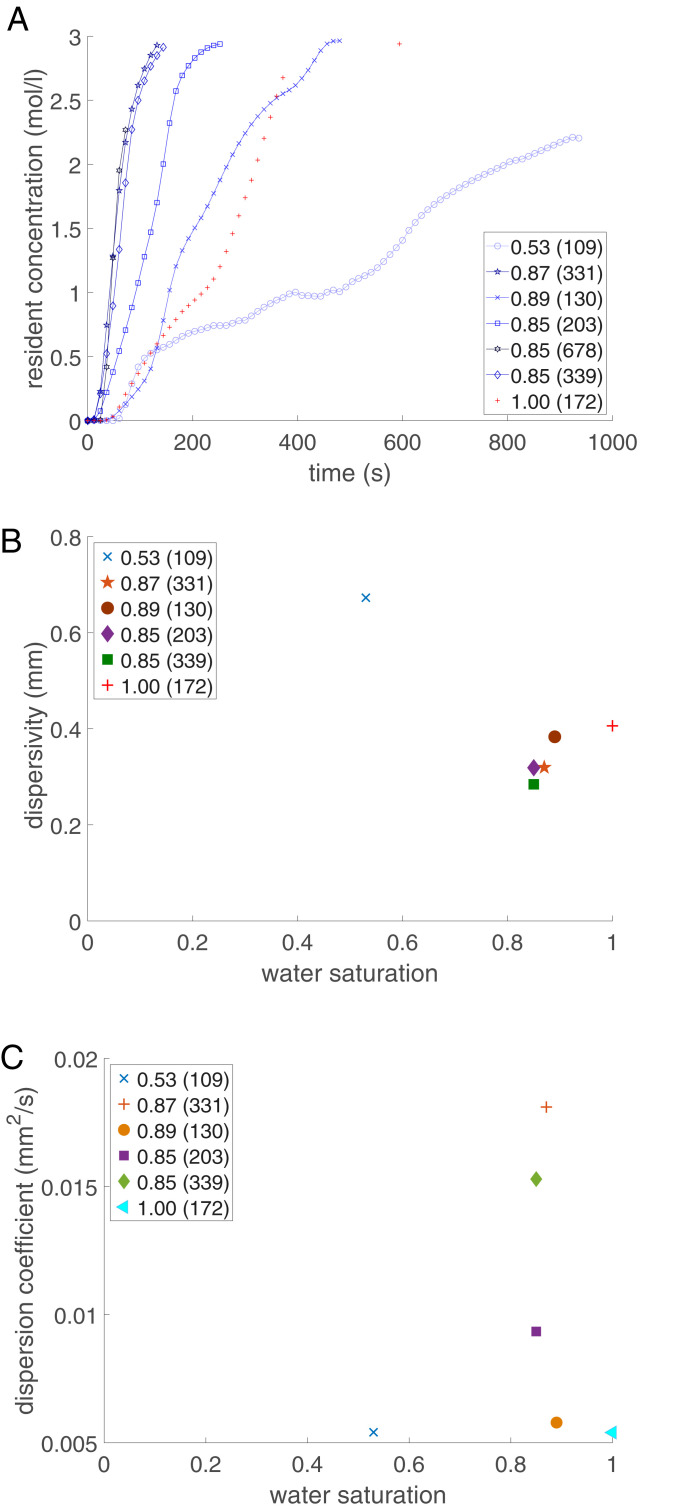
(*A*) Average resident concentration breakthrough curve for seven different water saturations with the Péclet number for each saturation shown in parentheses. (*B*) The relation between the dispersivity (mm) and water saturation for different water saturations and Péclet numbers. The Péclet number for each saturation case is shown in parentheses beside the saturation value. (*C*) Dispersion coefficient estimated using the time moment method for seven different water saturations. Each water saturation case has a different Péclet number shown within parentheses in the legend.

Due to the significant discrepancies in the transport time scales, which are spatially variable over the domain, the average resident concentration versus time can change dramatically as a function of the macroscale Péclet number and saturation as shown in Fig. 5*A*. At a water saturation of 0.53, the graph indicates a rapid change of the KI concentration for times below 100 s, and after that, the curve shows a slow change of the KI concentration toward the injection concentration of 3.0 mol/L. These two features of the breakthrough curve have already been reported in earlier experimental studies (10, 47–49). As the water saturation approaches unity, the average resident concentration versus time follows the Fickian transport (Fig. 5*A*, red plus symbol). This shows that as the saturation approaches unity, the volume of slow (stagnant) regions reduces, which results in a Fickian behavior, and this was observed for all cases with a water saturation larger than 0.80.

The non-Fickian concentration profiles cannot be fitted using a single advection–dispersion equation (50) to estimate the corresponding dispersion coefficient, as well as dispersivity. There are alternative methods such as using the MIM (22) and MRMT (23) models to estimate the dispersion coefficients. However, they may lead to an incorrect estimation of the fitted parameters due to the inherent assumptions in those models (25). As an alternative approach, spatial moments (51) and time moments (52) can be used to estimate the dispersion coefficient. In this study, the dispersion coefficient (D) was estimated using the time moment for continuous injection (53). In Fig. 5*B*, dispersivity for six different water saturation values is plotted against water saturation. Each water saturation case had a different macroscale Péclet number, which is shown in the parentheses (Fig. 5*B*). At similar water saturations (0.85), dispersivities are relatively similar despite the difference of macroscale Péclet number and the presence of the stagnant zone. This indicated that the hydrodynamic conditions do not influence dispersivity assuming that the saturation topologies between the two similar saturation cases are not significantly different. For a range of saturation values (0.53, 0.89, and 1.00) and for a relatively narrow range of Péclet numbers (109, 130, and 172), dispersivity decreased with the decrease of saturation, which clearly highlights the importance of the saturation topology in dispersion. Given that hydrodynamics should not influence the dispersivity values, the differences in the dispersivity occur due to the presence of stagnant (slow) and flowing (fast) regions, which leads to the overestimation of dispersivity at intermediate water saturation, which has also been reported in the literature (47, 48, 54).

## Conclusions

The transient solute transport in single-phase and steady-state two-phase flow through a porous medium was visualized. An experimental method based on synchrotron X-ray imaging facilities at the Diamond Light Source, United Kingdom, was used to image the concentration field at a time resolution of 6 s (3 s for imaging and 3 s for sample rotation) and a spatial resolution of 3.25 μm.

The unique research results enabled us to show that even at a single pore, the solute transport can be non-Fickian. As pore-scale simulations imply, this might be due to separation of streamlines and lack of mixing in pore bodies, a combination of short pore throats (small mixing length) and very slow streamlines near no-slip walls. The results clearly invalidate the common assumption of full mixing at pore bodies and pore throats as even after injection of 17 pore volumes at high Péclet numbers, the concentration distribution across a pore was not uniform.

The 4D sCT indicates that for low macroscopic Péclet numbers, solute transport under partially saturated conditions can adequately be approximated as Fickian, while for high macroscopic Péclet numbers, it shifts toward the non-Fickian transport. Such a phenomenon was observed on the pore scale with the condition that the saturation values are almost similar. These results provide visualized evidence based on the resident concentration in the system which is spatially and temporarily variable.

Saturation topology is a key characteristic in defining transport in unsaturated porous media. Results show that for a narrow range of saturation, the resident concentration profiles change dramatically from Fickian to non-Fickian, and the non-Fickian trend is most significant in the intermediate saturation case (0.53). Also dispersivity in the porous medium increases at the intermediate range of saturation, which is less than twice the dispersivity of the fully saturated porous medium.

The experimental method and results clearly show that there is a new horizon to obtain time-variable characterization of transport in porous media which will help identifying the gaps in fundamental knowledge and improving the existing theories.

## Materials and Methods

A cylindrical flow cell (55) with the size of 4.8 mm in diameter and 50 mm in height was filled with glass beads with the average size of 150 μm. The flow cell was first saturated with the nonwetting phase by injecting nine pore volumes of Fluorinert (ninefold of void space). Then, pure water was injected into the flow cell to create the partially saturated conditions. After establishing the steady-state saturation conditions, we injected nine pore volumes of potassium iodide (KI) aqueous solution with the concentration of 3.0 mol/L, at the rate of 0.3 μL/s. The injection of nine pore volumes of water solution of KI was used to ensure that the KI solution would be dispersed sufficiently in the water-occupied area. The transport process was repeated at the rate of 0.6, 0.9, 1.5, and 3.0 μL/s. Also, ranges of flow rate were used to establish difference steady-state saturation condition that measured as water saturation. The whole transport and displacement process were imaged by the sCT located in the beamline i12, Diamond Light Source, United Kingdom. After that, projections were reconstructed using a Diamond Light Source in-house Python-based reconstruction code, and as a result, the image with the total size of 6 mm × 6 mm × 3.5 mm at the voxel size of 3.25 μm × 3.25 μm × 3.25 μm was produced for every 6 s. Finally, the reconstructed image was filtered, segmented, and postprocessed using Avizo and MATLAB for network extraction, and pore scale modeling for flow field computation. Detailed methods are written in *SI Appendix*.

For the postprocessing of the resident concentration profiles to estimate the dispersivity, the method of time moments was used. The time moment for continuous injection is given as $D=\frac{M_{2}}{2z}{\left(\frac{z}{M_{1}}\right)}^{3}$, where $M_{2}$ is the second central moment, $M_{1}$ refers to the first central moment, and z is the length of the domain. The first central moment is defined as $M_{1}=\frac{\int_{0}^{\infty }t^{1}\frac{\delta C}{\delta t}dt}{\int_{0}^{\infty }t^{0}\frac{\delta C}{\delta t}dt}$, where δC is the concentration difference calculated by deducting the average resident concentration at time $t_{i}$ from the average resident concentration at time $t_{i-1}$, and δt is the time difference which is fixed at 6 s. The second central moment is defined as $M_{2}=\frac{\int_{0}^{\infty }t^{2}\frac{\delta C}{\delta t}dt}{\int_{0}^{\infty }t^{0}\frac{\delta C}{\delta t}dt}$. The time moment dispersion coefficient is as illustrated in *SI Appendix*, Fig. S4. Further to that, one can estimate the dispersivity using the time moment approach which is given by $B=\frac{M_{2}}{2z}{\left(\frac{z}{M_{1}}\right)}^{2}$.

## Supplementary Material

Supplementary File

## Data Availability

Segmented images have been deposited in Mendeley (DOI: 10.17632/cjw5hb7y8p.1).
