# Supplementary material for: Direct characterization of solute transport in unsaturated porous media using fast X-ray synchrotron microtomography
Source: Proc Natl Acad Sci U S A. 2020 Sep 8;117(38):23443–9. doi: 10.1073/pnas.2011716117 (PMC7519338; doi:10.1073/pnas.2011716117)
Supplement: Supplementary File [file pnas.2011716117.sapp.pdf]

# Supplementary Information for

## Direct Characterisation of Solute Transport in Unsaturated Porous Media using 4D X-ray Synchrotron Microtomography

**Sharul Hasan, Vahid Niasar*, Nikolaos K. Karadimitriou, Jose R. A. Godinho, Nghia T. Vo, Senyou An, Arash Rabbani, and Holger Steeb**

*** Vahid Niasar.**
**E-mail: vahid.niasar@manchester.ac.uk**

### This PDF file includes:

Supplementary text
Figs. S1 to S5
Table S1
References for SI reference citations

www.pnas.org/cgi/doi/10.1073/pnas.2011716117

## Supporting Information Text

## Extended Methodology

The latest advances in X-ray imaging technology have enabled the end-user to conduct experiments on the pore scale while directly imaging the porous domain. This allowed for the estimation of static properties, such as porosity and permeability of a rock (1), or investigate two-phase flow dynamics (2, 3). When it comes to solute transport specifically, the scarcity of experimental data mainly on imaging of transient solute transport in porous media has been pointed out (1, 4). To our knowledge this is the first study which shows the capability of fast, high-resolution X-ray imaging in the study of transport in saturated and unsaturated porous media. We employed synchrotron-based, x-ray microtomography (sCT) and captured the 3D concentration field in the porous domain every 6 seconds, at the spatial resolution of $3.25\mu$m. The flow cell was a cylinder with a length of 50mm, and by 4.8mm in diameter, filled with glass beads. The glass beads had a mean size of $150\mu$m. The fluids used were water and Fluorinert (FC-43), serving as the wetting and the non-wetting phase, respectively. Potassium Iodide (KI) which is soluble in water, served as a tracer for imaging transport within the water phase. More specifically, iodine ions are known to be an attenuation factor for X-rays. By reasonably assuming that 1 M of iodine ions come from the dissolution of 1 M of KI we will describe processes and effects with respect to KI concentration. All experiments were performed under flow-controlled conditions, and a back pressure of 75 psi to avoid bubble generation during the high-energy X-ray imaging. The detailed experimental procedure was as follows.

***Transport under Unsaturated Conditions.*** 4D sCT was used to image six experiments of solute transport under unsaturated conditions, and one under fully-saturated conditions. The information for each of the transport experiments is presented in Table S1. To establish a steady-state, two-phase flow experiment, first the sample was saturated with the non-wetting fluid

**Table S1. List of transport experiments imaged using 4D sCT.**

| Flow rate ($\mu$l/s) | Péclet number | Water saturation |
|---|---|---|
| 0.3 | 109 | 0.53 |
| 1.5 | 331 | 0.87 |
| 0.6 | 130 | 0.89 |
| 0.9 | 230 | 0.85 |
| 3.0 | 678 | 0.85 |
| 1.5 | 339 | 0.85 |
| 0.9 | 172 | 1.00 (single-phase) |

(Fluorinert FC-43). Then, water was injected at a fixed injection rate, which led to a given steady-state saturation in the imaged section, as shown in Table S1. After having established the steady-state saturation, a KI aqueous solution at the concentration of 3 mol/l was injected at the same rate as water injection to avoid any mobilisation of the wetting fluid. This allowed us to image transport within the wetting phase while the saturation topology was not changing with time. All tracer injection experiments continued for 9 pore volumes to ensure that all the water-filled pore space had been filled with the KI solution.

During transport of the KI solution, a tomographic dataset was taken using a fast high-resolution sCT at the beamline I12-JEEP (Joint Engineering, Environmental, and Processing), Diamond Light Source (5) at six seconds time intervals. Based on the injection rate of the KI solution, the macroscopic Péclet number was estimated as $Pe = \frac{Q}{SA} * \frac{L}{D}$ where $Q$ is the volumetric injection rate$(\mu m^3/s)$, $S$ is the water saturation, $A$ is the cross-sectional area of the flow cell $(\mu m^2)$, $L$ is the the length of the imaged section $(\mu m)$, and $D$ is the molecular diffusion coefficient $(\mu m^2/s)$. The Péclet number for each experiment is tabulated in Table S1.

For the single-phase transport case the imaging process was similar to the unsaturated transport, however, water was initially injected into the dry glass beads packing. Then, the KI solution was injected into the flow cell at the controlled injection rate of 0.9 $\mu$l/s. The tomographic datasets were reconstructed using the I12 in-house Python codes (6–9) and this was applied to both single-phase and unsaturated transport experiments. Figure S1 shows the 3D reconstructed image of the flow cell filled with Fluorinert, KI aqueous solution, and glass beads. The experimental setup including the pumps, flow cell and connections as well as the X-ray imaged section of the flow cell can be seen in Figure S1.

***Post-Processing and Visualisation.*** As the first step for post processing images which would serve as masks needed to be acquired; (i) the fully-saturated mask: a binary image with the value of one representing the void space and zero for the solid phase, (ii) the unsaturated masks: binary images for the steady-state, two-phase flow with the value of one representing water and zero representing Fluorinert and glass beads.

To create the mask of the void space, the 32-bit reconstructed images of the dry sample (flow cell only filled with glass beads) were converted to 8-bit grayscale images. This was necessary as a means to reduce the computation time during the image segmentation and filtering. The re-scaled images were then imported to Avizo (10). Right after that, an image filtering method called 3D anisotropic diffusion was applied to the imported images. As a result, the noise and artefacts generated by the x-rays were removed, and this resulted in the grayscale intensity of the imported images to follow a bi-modal distribution, as shown in Figure S2.

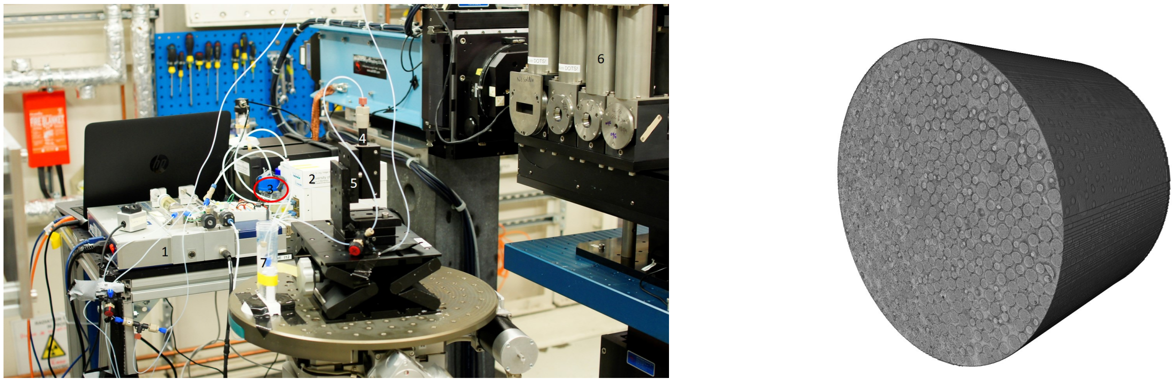


**(a)** **(b)**

**Fig. S1.** (a) The experimental setup. The numbering in the photo corresponds to the name of the equipment used during the transport experiment. Number 1 is the syringe pump, number 2 is the de-gasser which was used to remove the gas in the investigated fluid (water and aqueous solution of KI), number 3 is a 100ml container which contained either water or water solution of KI, number 4 is the flow cell (Peek cell) that contained the glass beads and the fluids, number 5 is the holder for the flow cell, number 6 is the x-ray detector, number 7 is a container that was used to collect the fluids the came out from the outlet. b) 3D visualisation of the flow cell generated by Diamond Light Source in-house reconstruction script. The flow cell had the dimensions of $4.8mm$ in diameter and $3.5$mm in length.

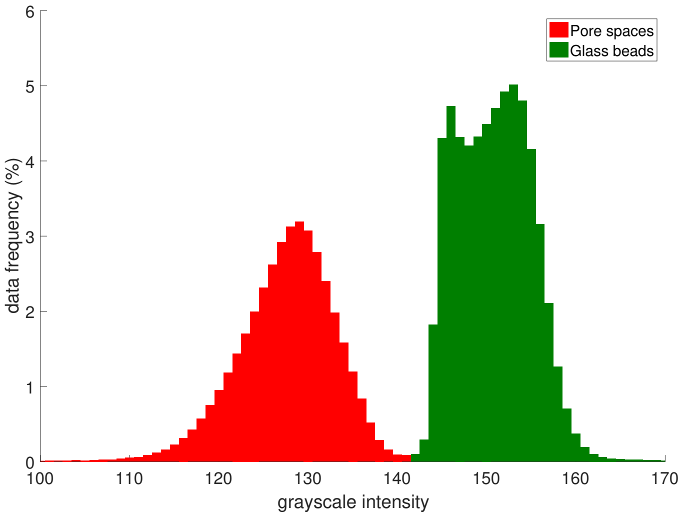


**Fig. S2.** Grayscale intensity distribution of a filtered 8-bit image. The red bar chart with grayscale intensity ranging between 100 and 140 is the pore spaces, while green bar chart with grayscale intensity ranging between 141 to 170 is the glass beads.

The first histogram with the peak grayscale value around 128 (Figure S2, red bar chart) represents the pore space, while the second histogram with the peak grayscale value around 155 (Figure S2, green bar chart) represents the glass beads. Finally, the mask of the pore space was generated by selecting the red bar chart with the grayscale value ranging between 100 and 140.

As the first step to create the mask of the water-filled space, the void space mask was applied to remove the solid space. Then, using local thresholds and edge detection(11), water and Fluorinert networks were segmented to identify the pore space occupied by Fluorinert. Using the steady-state, two-phase flow masks, the 3D snapshots of transport of the KI aqueous solution at each time step were generated. The above-mentioned steps were repeated for any other saturation case. Additionally, the segmented water phase was used to calculate the average resident concentration at each time step.

Figure S3 illustrates the 3D images of transport of the KI solution under unsaturated conditions at initial, intermediate and final situation. Under partially-saturated conditions, two fluids coexist in the porous media as shown in Figure S3. For better illustration, we assigned different colors to fluid phases; red represents Fluorinert, blue represents water with no KI concentration and green represents the high KI concentrations with the maximum of 3 mol/l.

***Network Extraction and Computation of the Velocity Field, and Local Concentration Gradient.*** In order to build the network model of the pore space based on the mask of the water phase, we utilized the watershed algorithm (12), earlier explained in (13). The resulted network was an interconnected structure of nodes and links which we call bodies and throats, respectively. To extract this network we started from the Euclidean distance transformation of the binarized geometry, which shows the distance of each voxel in the pore space to its nearest solid voxel. Then, by applying a Gaussian filter with the standard deviation of 2 voxels, we implicitly merged the local maximum values of the distance map to avoid over-segmentation of the pore space (14). Then, based on the watershed algorithm, we hypothetically started flooding the distance map from the largest values towards the smaller ones. This step-wise process, created a unique nucleus at the center of each pore body which dilated until it reaches the nucleus of an adjacent pore body. The locations at which two nuclei met were assumed as ridge lines and represented the narrowest section of the pathway between two pore bodies, which is called a pore throat. More details of the process are described in (12). In addition, considering the large size of the tomography images, we hired a domain decomposition approach to improve the computational efficiency of the pore network extraction by dividing the large structures into 8 sub-domains and merge them back after processing (15). The methodology for network extraction was repeated for all saturation cases.

The extracted pore geometry and topology was then imported into a single phase flow simulator (16) to compute the local pore-scale velocity. The local pore-scale velocity was computed by solving the continuity equation and assuming Hagen-Poiseuille flow. The detailed formulation for solving the continuity equation can be found in the previous study (16). After that, the local Péclet number was computed using the following formulation. $Pe_{ij} = \frac{v_{ij} r_{ij}}{D_m}$ where the $v_{ij}$ is the computed local pore-scale velocity of the pore throat $ij$, $r_{ij}$ is the radius of the pore throat $ij$, and $D_m$ is the molecular diffusion. The above steps were repeated for the other saturation cases.

To be able to convert the grayscale values to concentration values, we carried out several calibration experiments, where a dry glass-bead sample was flooded with a KI aqueous solution of a known concentration until a steady state (spatially uniform concentration) was reached. Then, based on the mean grayscale pixel value of the steady-state concentration and the known concentration, the correlation curve between the grayscale intensity and KI concentration was constructed as shown in Figure S4.

According to previous studies, under partially-saturated conditions and for a given fluid, two different zones named as stagnant and flowing were identified in experiments (17, 18) and simulations(19, 20). Given the 3D network of the water phase

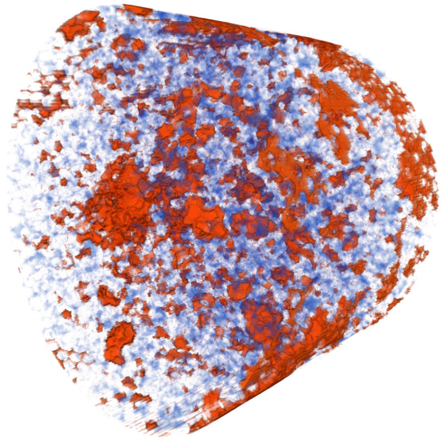

**(a)**

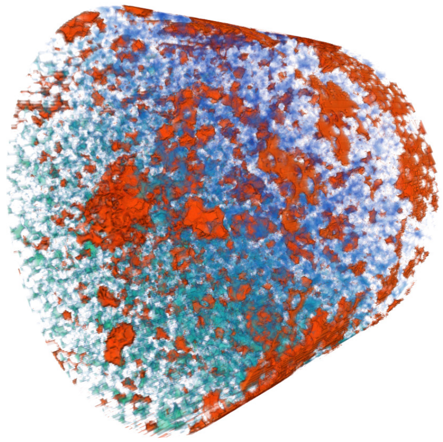

**(b)**

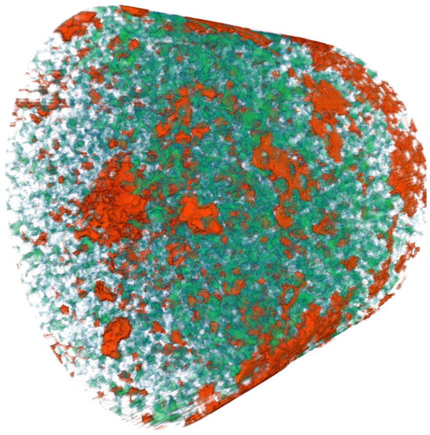

**(c)**

**Fig. S3.** KI transport under partially-saturated conditions at (a) initial condition, (b) transient condition at the average concentration of 1.5mol/l, and (c) steady-state condition. Red represents Fluorinert, blue to green spectrum represents the KI concentration from zero to 3 mol/l.

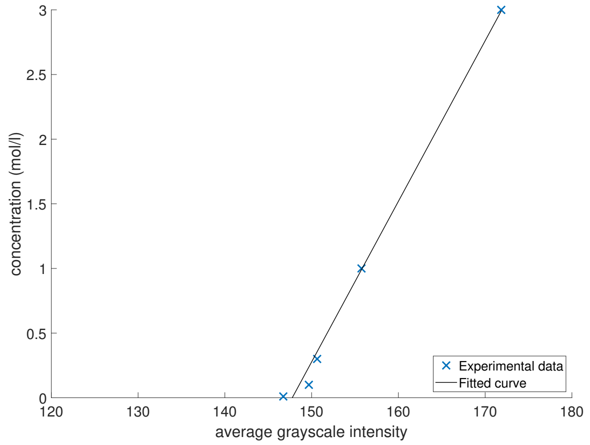


**Fig. S4.** The relation between the KI concentration in molarity and average grayscale value. The 'x' symbol is the experimental data and the solid line is the linear fitted curve.

in a water-wet sample, a sharp contrast between the intensities of the KI solution in close distances were not easily identified. However, we applied a local gradient operator to the concentration field to identify whether there were significant differences between the statistical distribution of the local concentration differences. The local concentration difference was calculated as $\max|c_i - c_j|$; with $c_i$ and $c_j$ being the concentrations in two neighboring voxels belonging to the water network.

Also, we employed the computational approach to identify whether there were significant discrepancies in the transport time scales in the water network. By solving the velocity field in the water-filled network, we identified the statistical distribution of the velocity, and the histogram of the velocity distribution were generated.

***Streamline simulations.*** To further investigate the cause of very slow mixing in pores, we conducted high-resolution lattice Boltzmann simulations to solve the velocity field. For the case of saturation of 0.89, we performed GPU-accelerated volumetric lattice Boltzmann method (VLBM) (21, 22). Since running the simulations on the full size image of the experiment was not computationally feasible, the image was cropped to the size of 400×400×400 voxels with the resolution 3.25 $\mu$m/pixel (Figure S5a). We carried out the calculation on an NVIDIA Tesla P100 GPU card, which has 3584 CUDA cores with 1190 MHZ clock frequency and 16GB of global memory. With GPU-based parallelization, one single-phase flow simulation was done in 10 minutes.

For an improved clarify, Figure S5b shows the streamlines is a very small section of the simulated domain. The streamlines clearly show that there is not a complete mixing at pore bodies. As the experimental results of the present 3D study and former 2D micromodel studies(23), there is not a fully mixing condition within pore bodies. Even in pore throats the streamlines have very different trajectories at different velocities, which leads to a very non-uniform distribution of concentration across the cross section.

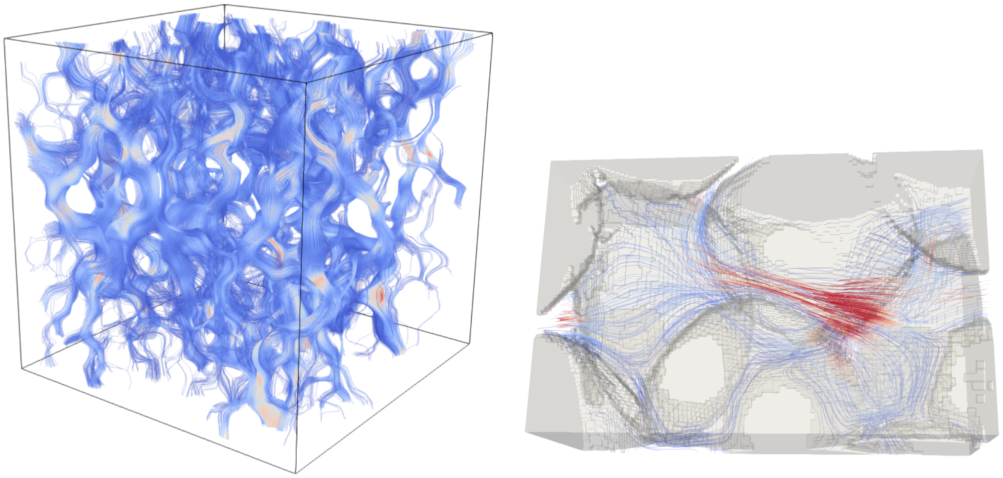

**Fig. S5.** Volumetric Lattice Boltzmann-based streamline simulation, a) $400^3$ voxel image, b) a pore-scale presentation of streamlines that shows non-uniform distribution of velocity.

## References


1. Wildenschild D, Sheppard AP (2013) X-ray imaging and analysis techniques for quantifying pore-scale structure and processes in subsurface porous medium systems. *Advances in Water Resources* 51:217–246.
2. Berg S, et al. (2013) Real-time 3d imaging of haines jumps in porous media flow. *Proceedings of the National Academy of Sciences* 110(10):3755.
3. Pak T, Butler IB, Geiger S, van Dijke MIJ, Sorbie KS (2015) Droplet fragmentation: 3d imaging of a previously unidentified pore-scale process during multiphase flow in porous media. *Proceedings of the National Academy of Sciences* 112(7):1947.
4. Cnudde V, Boone MN (2013) High-resolution x-ray computed tomography in geosciences: A review of the current technology and applications. *Earth-Science Reviews* 123:1–17.
5. Drakopoulos M, et al. (2015) I12: the joint engineering, environment and processing (jeep) beamline at diamond light source. *Journal of Synchrotron Radiation* 22(3):828–838.
6. Ramachandran GN, Lakshminarayanan AV (1971) Three-dimensional reconstruction from radiographs and electron micrographs: Application of convolutions instead of fourier transforms. *Proceedings of the National Academy of Sciences* 68(9):2236.
7. Vo NT, Drakopoulos M, Atwood RC, Reinhard C (2014) Reliable method for calculating the center of rotation in parallel-beam tomography. *Optics Express* 22(16):19078–19086.
8. van Aarle W, et al. (2016) Fast and flexible x-ray tomography using the astra toolbox. *Optics Express* 24(22):25129–25147.
9. Vo NT, Atwood RC, Drakopoulos M (2018) Superior techniques for eliminating ring artifacts in x-ray micro-tomography. *Optics Express* 26(22):28396–28412.
10. Amira-Avizo (2017) Amira-avizo for three-dimensional visualisation.
11. Canny J (1986) A computational approach to edge detection. *Pattern Analysis and Machine Intelligence, IEEE Transactions on* PAMI-8:679–698.
12. Rabbani A, Jamshidi S, Salehi S (2014) An automated simple algorithm for realistic pore network extraction from micro-tomography images. *Journal of Petroleum Science and Engineering* 123:164–171.
13. Joekar Niasar V, Hassanizadeh SM, Pyrak-Nolte LJ, Berentsen C (2009) Simulating drainage and imbibition experiments in a high-porosity micromodel using an unstructured pore network model. *Water Resources Research* 45(2).
14. Gostick JT (2017) Versatile and efficient pore network extraction method using marker-based watershed segmentation. *Physical Review E* 96(2):023307.
15. Rabbani A, Mostaghimi P, Armstrong RT (2019) Pore network extraction using geometrical domain decomposition. *Advances in Water Resources* 123:70 – 83.
16. Babaei M, Joekar-Niasar V (2016) A transport phase diagram for pore-level correlated porous media. *Advances in Water Resources* 92:23–29.
17. Karadimitriou NK, Joekar-Niasar V, Babaei M, Shore CA (2016) Critical role of the immobile zone in non-fickian two-phase transport: A new paradigm. *Environmental Science & Technology* 50(8):4384–4392.
18. Karadimitriou NK, Joekar-Niasar V, Brizuela OG (2017) Hydro-dynamic solute transport under two-phase flow conditions. *Scientific Reports* 7(1):6624.
19. Aziz R, Joekar-Niasar V, Martinez-Ferrer P (2018) Pore-scale insights into transport and mixing in steady-state two-phase flow in porous media. *International Journal of Multiphase Flow* 109:51–62.
20. Hasan S, Joekar-Niasar V, Karadimitriou NK, Sahimi M (2019) Saturation dependence of non-fickian transport in porous media. *Water Resources Research* 55(2):1153–1166.
21. An S, Yu HW, Wang Z, Kapadia B, Yao J (2017) Unified mesoscopic modeling and gpu-accelerated computational method for image-based pore-scale porous media flows. *International Journal of Heat and Mass Transfer* 115:1192–1202.
22. An S, Yu HW, Yao J (2017) Gpu-accelerated volumetric lattice boltzmann method for porous media flow. *Journal of Petroleum Science and Engineering* 156:546–552.
23. Oostrom M, Mehmani Y, Romero-Gomez P, , et al. (2016) Pore-scale and continuum simulations of solute transport micromodel benchmark experiments. *Comput Geosci* 20:857–879.
